# Lipopolysaccharide preconditioning of adipose-derived stem cells improves liver-regenerating activity of the secretome

**DOI:** 10.1186/s13287-015-0072-7

**Published:** 2015-04-14

**Authors:** Sang Chul Lee, Hye Jin Jeong, Sang Kuon Lee, Say-June Kim

**Affiliations:** Department of Surgery, Daejeon St. Mary’s Hospital, College of Medicine, The Catholic University of Korea, Daeheung-dong 520-2, Joong-gu, Daejeon Republic of Korea

## Abstract

**Introduction:**

Growing recognition of paracrine mechanisms in stem cell plasticity has resulted in considerable interest in stem cell-derived secretome. The aim of this study was to investigate the effects of lipopolysaccharide (LPS) preconditioning on the composition and hepatic regenerative activity of adipose-derived stem cell (ASC) secretome.

**Methods:**

Conditioned medium (CM) and LPS-CM were obtained after culturing human ASCs without or with low-dose LPS (0.5 ng/mL) for 24 hours. Untreated and thioacetamide-treated mouse AML12 hepatocytes were incubated for 24 hours with the control medium, LPS (0.5 ng/mL), CM, and LPS-CM and then cell viabilities were compared. CM and LPS-CM were also intravenously administered to partially hepatectomized mice, and their effects on liver regeneration were assessed by using liver weight measurements, immunohistochemistry, and Western blotting.

**Results:**

In the *in vitro* experiments, LPS preconditioning of ASCs enhanced the mRNA expression levels of interleukin-6 (IL-6), tumor necrosis factor-alpha (TNF-α), hepatocyte growth factor, and vascular endothelial growth factor, which evoke inflammatory response or liver regeneration. LPS-CM significantly promoted thioacetamide-damaged AML12 cell viability compared with CM-incubated cells and the control cells (77%, 69%, and 65% *P* <0.05). In the *in vivo* experiment, LPS-CM infusion into the partially hepatectomized mice significantly reduced serum IL-6 and TNF-α levels compared with the other groups (*P* <0.05) on days 1 and 2 after partial hepatectomy. Moreover, LPS-CM infusion enhanced liver regeneration (based on the liver weight changes at day 7 after partial hepatectomy, 3.73% versus 3.22% in the CM group; *P* <0.05) and significantly reduced the elevated serum levels of aspartate transaminase and alanine transaminase (at day 1, *P* <0.05).

**Conclusions:**

Our results suggest that LPS preconditioning effectively stimulates ASCs to produce the secretome beneficial to hepatic regeneration. Thus, optimizing ASC secretome profile by LPS preconditioning could be a promising approach to treat liver diseases by using stem cells.

## Introduction

Over the last decades, mesenchymal stem cells (MSCs) have been extensively studied with regard to their potential implications in regenerative medicine. MSCs have demonstrated several advantages, including higher availability, functional plasticity, and lower immunogenicity [1]. Among the various sources of MSCs, adipose tissue is gaining more and more interest because large amounts of adipose tissue-derived stem cells (ASCs) can be obtained by less invasive methods and thus they are considered major candidates for future regenerative medicine [2]. However, despite the encouraging results of preclinical studies using ASCs, its clinical application is still hindered by several limitation factors, including poor growth kinetics, early senescence, and genetic instability during *in vitro* expansion, as well as the possibility of malignant transformation [3-5].

Meanwhile, increasing evidence has been accumulating in support of the notion that the principal mechanism of stem cell-based therapeutic activity is secretome-related [1,6-9]. Thus, an exclusive use of MSC-secreted molecules rather than the cells per se can be one way to circumvent the limitations of cell-based therapy while maintaining its advantages. The total set of molecules secreted or surface-shed by stem cells is generally referred to as secretome. The secretome includes bioactive peptides, including cytokines, chemokines, and growth factors [6,9-11]. These soluble factors are released from ASCs either solitarily or in the form of extracellular vesicles (EVs). The EVs include exosome (50 to 100 nm in diameter) and the larger microvesicle (50 to 100 nm in diameter) [12]. EVs are particularly important because they have the ability to transfer proteins and functional genetic material such as RNA to other cells [13-15]. Therefore, the use of these cell-free products may indeed represent an alternative to the therapies based on cell transplantation.

Improving liver regenerative ability after partial hepatectomy (PH) or living donor liver transplantation has been a primary concern, especially for patients with cirrhosis or other functional liver impairment. Recent research indicated that the intravenous administration of MSCs could enhance liver regenerative ability in the murine models of either toxin-induced hepatic failure or PH [16-20]. Moreover, the isolated administration of secretome, instead of MSCs themselves, has shown the equivalent liver regenerative ability, demonstrating the paracrine effect of MSCs [21-23]. The spectrum of regulatory factors making the secretome can be shaped by genetic engineering of ASCs or by modifying their physical or chemical environment (that is, reconditioning) or both. It is well established that the composition of ASC secretome is significantly dependent on culturing conditions; therefore, the optimization of culture methodology can be the easiest way to obtain a secretome with high therapeutic potential.

It has been suggested that MSC stimulation with Toll-like receptor 4 (TLR4) agonists polarizes human MSCs toward a pro-inflammatory phenotype but that MSC stimulation with TLR3 agonists polarizes them toward an immunosuppressive phenotype [24]. Lipopolysaccharide (LPS) is a representative TLR4 agonist and induces MSCs to release pro-inflammatory cytokines, including interleukin-1-beta (IL-1β), IL-6, IL-8, IL-12, type I interferons (IFNs), and tumor necrosis factor-alpha (TNF-α) [25,26]. Moreover, MSC pretreatment with low, non-toxic LPS concentrations prior to transplantation has been shown to increase resistance to tissue damage in various organs [9,26-28]. Considering these findings, we suppose that LPS preconditioning of ASCs would harness the ASCs to generate the secretome with higher liver regenerative ability. In this study, we attained the ASC secretome under the LPS preconditioning and evaluated its effectiveness in terms of liver regeneration, anti-inflammation, and organ restorative capacity in both *in vitro* and *in vivo* models. These results could help determine the optimal culture conditions for obtaining a high-yield secretome that is most beneficial for liver recovery.

## Methods

### Adipose tissue-derived stem cell culture

Human ASCs (third passage) were kindly donated by Hurim BioCell Company (Seoul, Korea). The institutional review board of Hurim BioCell Company (registration number in Korea Center for Disease Control & Prevention: 1-700069-B-N-01) approved the attainment of human ASCs, and informed consents were obtained from all of the patients. Cultured ASCs have been shown to display MSC phenotype: they express the MSC marker CD90 and do not express hematopoietic markers CD31 and CD34 [29]. ASCs were cultured and ASC secretome was prepared the way we previously described [29].

### Preparation of lipopolysaccharide-conditioned medium

ASCs were grown in 100-mm cell dishes (Corning Glass Works, Corning, NY, USA). After reaching 70% to 80% confluence, 5.0 × 10^5^ ASCs were cultured in 5 mL of serum-free low-glucose Dulbecco’s modified Eagle’s medium (DMEM) (Thermo Scientific, Hemel Hempstead, UK) with or without LPS in low concentration (0.5 ng/mL; Sigma-Aldrich, St. Louis, MO, USA) for 24 hours. In a variety of experiments including intravenous administration of MSCs, 0.1 mL of 1 × 10^5^ to 1.0 × 10^6^ MSCs has been used for an injection into a mouse [17,30-32]. In our protocol, the secretome from 5.0 × 10^5^ ASCs was cultured in 5 mL of DMEM. Therefore, to obtain the equivalent amount (0.1 mL) of secretome, the conditioned media were concentrated 25-fold by using ultrafiltration units with a 3-kDa-molecular-weight cutoff (Amicon Ultra-PL 3; Millipore, Bedford, MA, USA) after stimulation with or without LPS. From here on, LPS-CM and CM refer to the 25-fold concentrated conditioned media which had been obtained from ASCs after stimulation with or without LPS for 24 hours.

### Intravenous administration of conditioned medium and lipopolysaccharide- conditioned medium in partially hepatectomized mice

We designed a case–control study to assess the effect of intravenous administration of CM and LPS-CM by using 8-week-old male BALB/c mice (Damool Science, Daejeon, Korea). This study was approved by the Institutional Animal Care and Use Committee of the Clinical Research Institute at Daejeon St. Mary’s Hospital at the Catholic University of Korea (institutional review board #CMCDJ-AP-2014-007). The animals were housed under pathogen-free conditions and received humane care in accordance with the criteria outlined in the Guide for the Care and Use of Laboratory Animals, which was prepared by the National Academy of Sciences (National Institutes of Health publication 86–23, 1985 revision). Approximately 70% PH was performed under tiletamine-zolazepam sedation (30 mg/kg intraperitoneal Zoletil; Virbac, Nice, France) [33]. Liver regeneration was expressed as the ratio (percentage) of liver weight to body weight (LW/BW) [34-36]. Within 1 hour after PH, the mice were divided into four experimental groups and were intravenously administered 0.1 mL of low-glucose DMEM (control group), 0.5 ng/mL LPS in low-glucose DMEM (LPS group), CM (CM group), and LPS-CM (LPS-CM group).

Each group included 25 mice (total 100 mice), which were separated into two subgroups: those used to evaluate consecutive alterations in serology, such as serum levels of aspartate transaminase (AST) and alanine transaminase (ALT) (n = 5), and those that were sacrificed to obtain sera for enzyme-linked immunosorbent assay (ELISA) and histologic specimens at specific time points (1, 2, 3, and 7 days) after material administration (n = 20).

### Real-time quantitative polymerase chain reaction

Total ASC RNA was extracted by using TRIzol reagent (Invitrogen, part of Thermo Fisher Scientific, Waltham, MA, USA) in accordance with the instructions of the manufacturer. Reverse transcription was performed with 1 μg of RNA, random primers, and M-MLV Reverse transcriptase (Promega, Madison, WI, USA). The primers used for SYBR green quantitative real-time polymerase chain reaction (qRT-PCR) were the following: IL-6 forward 5′-CACACAGACAGCCACTCACC-3′ and reverse 5′-TTTTCTGCCAGTGCCTCTTT-3′; TNF-α forward 5′-AACCTCCTCTCTGCCATCAA-3′ and reverse 5′-GGAAGACCCCTCCCAGATAG-3′; vascular endothelial growth factor (VEGF) forward 5′-TCTTCAAGCCATCCTGTGTG-3′ and reverse 5′-ATCTGCATGGTGATGTTGGA-3′; hepatocyte growth factor (HGF) forward 5′-TGCTGTCCTGGATGATTTTG-3′ and reverse 5′-AGTGTAGCCCCAGCCATAAA-3′; and glyceraldehyde 3-phosphate dehydrogenase (GAPDH) forward 5′- GCACCGTCAAGGCTGAGAAC-3′ and reverse 5′-TGGTGAAGACGCCAGTGGA-3′. The reaction was performed by using an Applied Biosystems 7500 Fast Real-Time PCR System (Life Technologies, Carlsbad, CA, USA) equipped with a 96-well optical reaction plate reader. The expression levels of the target genes were calculated by using the comparative threshold cycle method and normalized to *GAPDH* as a house-keeping gene. The data are presented as the mean ± standard deviation from three independent experiments.

### Enzyme-linked immunosorbent assay

In an *in vitro* experiment, CM and LPS-CM were collected from each dish and centrifuged at 1,500 revolutions per minute (rpm) for 5 minutes, and human IL-6 release was measured by using an ELISA kit (eBioscience Inc., San Diego, CA, USA) in accordance with the instructions of the manufacturer. In an *in vivo* experiment, concentrations of mouse IL-6 and TNF-α were determined in the serum of partially hepatectomized mice at 1, 2, and 3 days after the injection of control medium, LPS, CM, and LPS-CM by using ELISA kits (eBioscience Inc. and Biolegend Inc., San Diego, CA, USA).

### Cell viability assay

A non-tumorigenic mouse hepatocyte cell line AML12 (CRL-2254) was purchased from American Type Culture Collection (Manassas, VA, USA) and maintained in DMEM/Ham F12 (Invitrogen) supplemented with 10% fetal bovine serum (Invitrogen), 1× insulin-transferrin-selenium-G supplement (Invitrogen), 40 ng/mL dexamethasone (Sigma-Aldrich), and 100 ng/mL amphotericin B (Invitrogen). AML12 cells were plated 1 × 10^4^ cells per well in 96-well plates and allowed to adhere overnight. Thereafter, CM or LPS-CM was added, followed by incubation for 24 hours at 37°C. In the TLR4 inhibition test, prior to incubation with CM or LPS-CM, cells were pre-treated with TLR4 inhibitor TAK-242 (1 μM; Calbiochem, La Jolla, CA, USA) or dimethyl sulfoxide (Sigma-Aldrich) as vehicle control for 1 hour. Then, hepatocyte toxicity was induced by maintaining AML12 cells in the presence of 50 mM thioacetamide (TAA) (Sigma-Aldrich). Normal and TAA-treated AML12 cells were incubated with the control medium, LPS (0.5 ng/mL), CM, and LPS-CM, and cell viability was examined by using the Ez-Cytox cell viability assay kit (Daeil Lab Service Co., Ltd, Seoul, Korea).

### Immunohistochemical analysis of Ki67 expression

Paraffin-embedded tissue sections were deparaffinized in xylene and rehydrated in a graded series of alcohol. The antigen was retrieved by heating the sample with 0.01 M citrate buffer (pH 6.0) in an autoclave (CHS-ACCE-860; JW Pharmaceutical, Seoul, Korea) at 121°C for 5 minutes. The sections were placed in 3% hydrogen peroxide for 5 minutes to inactivate the endogenous peroxidase, blocked for 10 minutes with protein block serum-free solution (Dako, Glostrup, Denmark), and incubated overnight at 4°C with the primary rabbit polyclonal antibody against Ki67 nuclear antigen (1:300; Abcam, Inc., Cambridge, MA, USA) to detect proliferating cells. The slides were treated with a biotinylated secondary antibody for 30 minutes at room temperature, followed by streptavidin-horseradish peroxidase (HRP) and 3,3′-diaminobenzidine solution (all from Dako) for another 10 minutes at room temperature, and counter-stained with hematoxylin.

### Western blot analysis

The effects of CM and LPS-CM on hepatotoxicity were assessed in both *in vitro* (TAA-treated AML12 cells) and *in vivo* (partially hepatectomized mice) experiments by Western blot analysis. TAA-treated AML12 cells incubated with DMEM, LPS, CM, and LPS-CM were lysed by using the EzRIPA Lysis kit (Atto Corporation, Tokyo, Japan) and were centrifuged at 12,000 rpm for 15 minutes, and the supernatants were collected. Liver tissues were lysed in lysis buffer (Roche Applied Science, Indianapolis, IN, USA). Protein concentration in the lysates was determined by using the Bradford reagent (Bio-Rad Laboratories, Hercules, CA, USA). Equal amounts of protein (30 μg) per well were separated by SDS-PAGE and electro-transferred onto nitrocellulose membranes, which were blocked with 5% non-fat milk at room temperature for 1 hour, and incubated with primary antibodies (1:1,000 dilution) against proliferating cell nuclear antigen (PCNA), phospho-signal transducer and activator of transcription 3 (p-STAT3) (Tyr^705^), STAT3, and β-actin at 4°C overnight and with HRP-conjugated secondary anti-rabbit and anti-mouse IgG (1:2,000 dilution) for 1 hour at room temperature (all from Cell Signaling, Beverly, MA, USA). Specific immune complexes were detected by using Western Blotting Plus Chemiluminescence Reagent (Millipore).

### Assessment of liver functions

Blood samples were obtained from each mouse and centrifuged for 10 minutes at 10,000 rpm, and the serum was collected. Concentrations of transaminases AST and ALT, the indicators of liver injury, were measured by using the Idexx VetTest Chemistry Analyzer (Idexx Laboratories Inc., Westbrook, ME, USA).

### Statistical analysis

All data were analyzed by using SPSS 11.0 software (SPSS Inc., Chicago, IL, USA) and are presented as the mean ± standard deviation. Statistical comparisons among groups were performed by using the Kruskal-Wallis and Mann–Whitney *U* tests; the Mann–Whitney *U* test was used to compare mean values between two groups, and the Kruskal-Wallis test was used to compare mean values among three or more groups. Probability values of *P* less than 0.05 were considered statistically significant.

## Results

### Effects of lipopolysaccharide preconditioning on mRNA expression and secretion of pro-inflammatory cytokines

**Figure 1:**
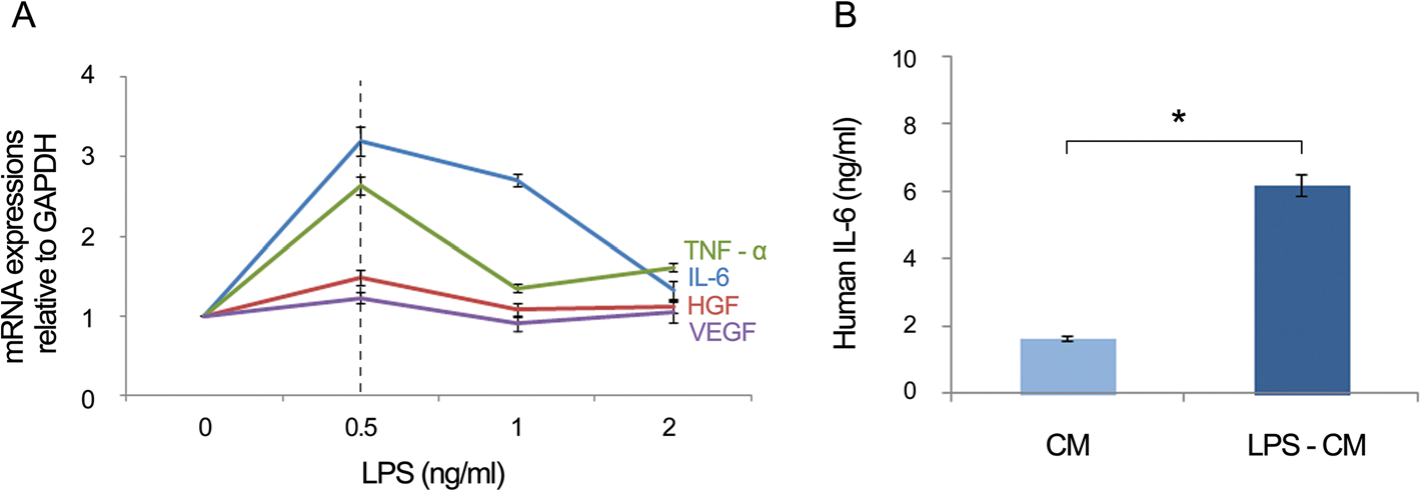
Effects of lipopolysaccharide (LPS) preconditioning of adipose tissue-derived stem cells (ASCs) on mRNA expression and secretion of pro-inflammatory cytokines. **(A)** ASCs were cultured in serum-free low-glucose Dulbecco’s modified Eagle’s medium with or without LPS in low concentration (0.5 ng/mL) for 24 hours and analyzed for mRNA expression of interleukin-6 (IL-6), tumor necrosis factor-alpha (TNF-α), hepatocyte growth factor (HGF), and vascular endothelial growth factor (VEGF) by quantitative polymerase chain reaction. **(B)** Conditioned media (CM and LPS-CM) were collected, concentrated 25-fold, and analyzed for IL-6 concentration by enzyme-linked immunosorbent assay. Values represent mean ± standard deviation of four independent experiments (*P* <0.05). *P <0.05. CM, conditioned medium; GAPDH, glyceraldehyde 3-phosphate dehydrogenase.

IL-6 and TNF-α are representative pro-inflammatory cytokines [37], and HGF and VEGF are well-known hepatic mitogens [34,38]. Human ASCs after six or more passages were incubated with increasing concentrations of LPS for 24 hours, and mRNA expression of these mediators was determined by quantitative PCR. The levels of these markers were increased at 0.5 ng/mL LPS but thereafter decreased (Figure 1A), indicating that low-dose LPS preconditioning upregulated mRNA expression of these mediators. Next, we directly measured human IL-6 concentrations in the human ASCs which had been maintained with or without 0.5 ng/mL LPS (LPS-CM and CM) for 24 hours. IL-6 levels were significantly higher in LPS-CM than in CM (6.19 versus 1.65 ng/mL; *P* <0.05), demonstrating that low-dose LPS triggers IL-6 secretion by ASCs (Figure 1B).

### Effects of conditioned medium and lipopolysaccharide-conditioned medium on normal and thioacetamide-treated AML12 cells

**Figure 2:**
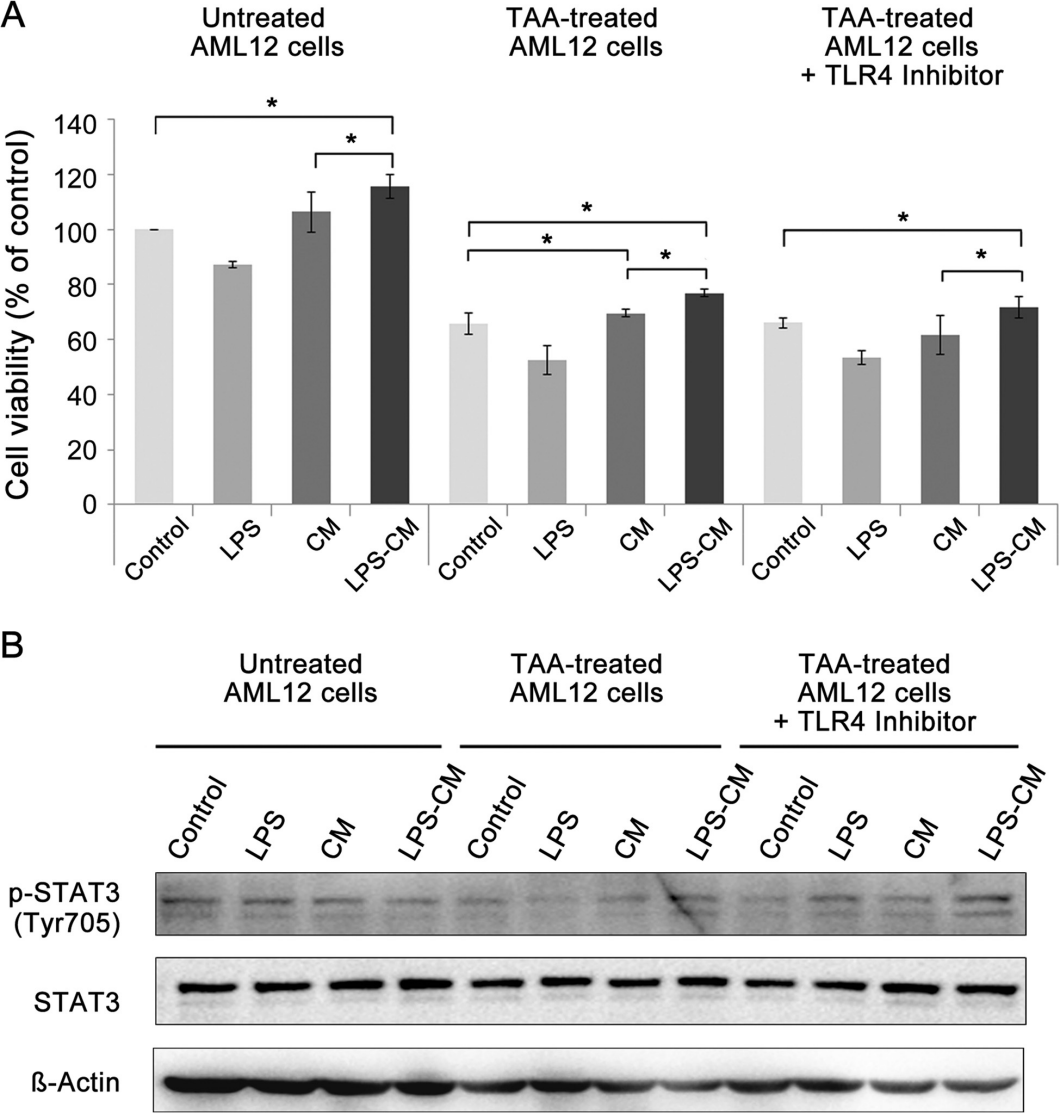
Effects of conditioned medium (CM) and lipopolysaccharide (LPS)-CM on the viability of normal and hepatotoxic agent-treated mouse hepatocytes. **(A)** Untreated and 50 mM thioacetamide (TAA)-treated and TAA + TLR4 inhibitor (TAK-242) AML12 cells were incubated for 24 hours with the control medium, LPS (0.5 ng/mL), CM, and LPS-CM and cell viabilities were determined by using the Ez-Cytox cell viability assay. Values represent mean ± standard deviation of four independent experiments (*P* <0.05). **(B)** The expressions of phospho-signal transducer and activator of transcription 3 (p-STAT3) and STAT3 in AML12 cells treated as above were analyzed by Western blotting. *P <0.05. TLR4, Toll-like receptor 4.

We investigated the effects of CM and LPS-CM on the proliferation of mouse hepatocyte AML12 cells. Untreated and TAA-treated mouse AML12 cells were cultured with control medium, LPS, CM, and LPS-CM and then cell viabilities were determined. In both untreated and TAA-treated AML12 cells, LPS-CM groups showed the highest viability (115.8% and 77%) and the CM groups were second (106.5% and 69.6%, respectively) (*P* <0.05) (Figure 2A). Moreover, we performed an experiment by using TLR4 inhibitor, TAK-242, to obtain the clear-cut evidence that the effectiveness of LPS-CM is driven not by LPS but by LPS-CM. LPS signaling is mediated by TLR4 and involves the coordinated production of a multitude of inflammatory mediators [24]. Therefore, to block LPS signaling, TAK-242 was supplemented to the TAA-treated AML12 cells which had been cultured with control medium, LPS, CM, and LPS-CM. TAK-242 treatment did not result in significant changes in either the LPS or the LPS-CM group, suggesting no relationship between LPS-CM effectiveness and LPS/TLR4 signaling.

It is well known that IL-6/STAT3 signaling plays a crucial role in the mitogenic response in the liver [39]. The expression levels of p-STAT3 were significantly reduced in TAA-treated compared with untreated AML12 cells. However, CM reversed the reduction, whereas LPS-CM caused further increase in the p-STAT3 expression. Again, TAK-242 treatment did not result in significant changes of the p-STAT3 expression in the LPS-CM group (Figure 2B).

### Effect of lipopolysaccharide-conditioned medium on the secretion of pro-inflammatory cytokines in partially hepatectomized mice

**Figure 3:**
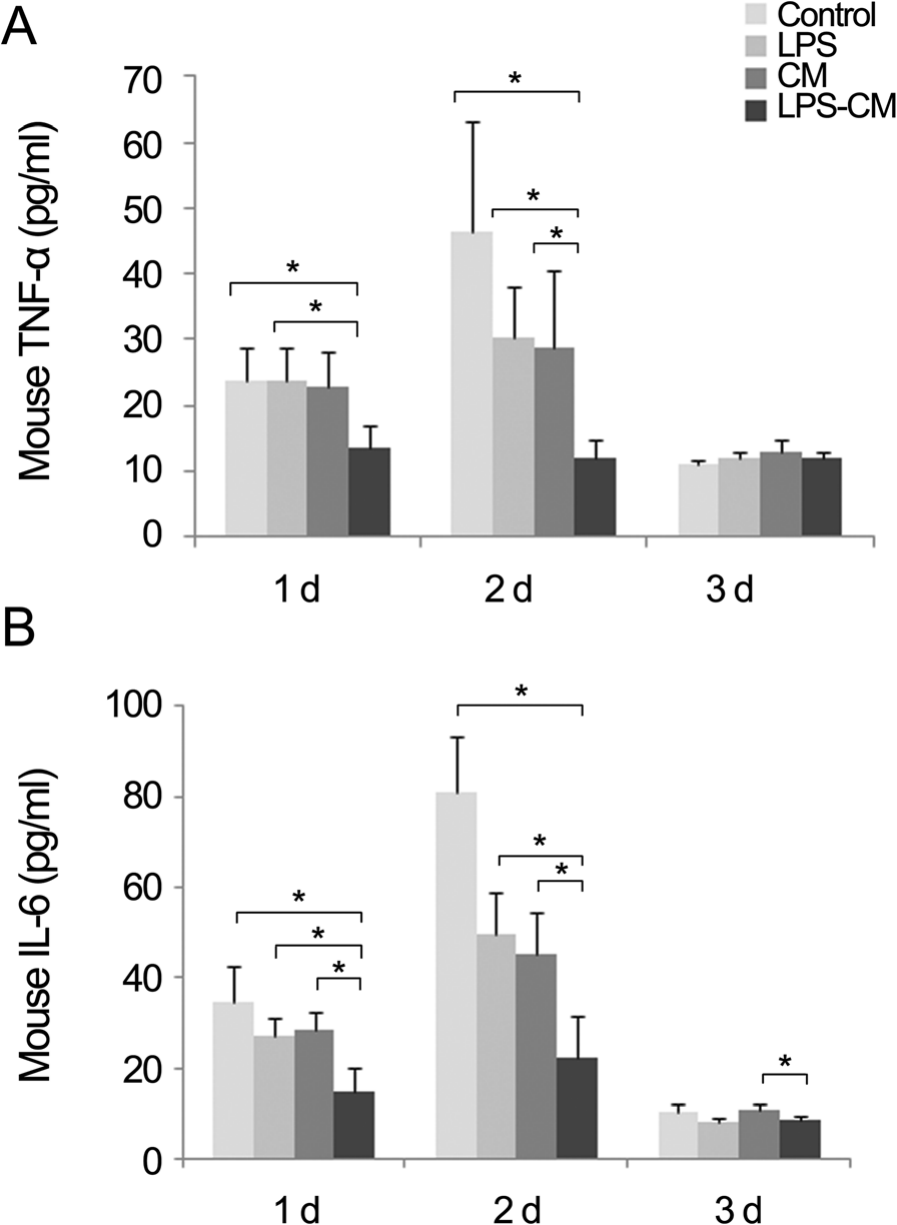
Serum concentrations of tumor necrosis factor-alpha (TNF-α) and interleukin-6 (IL-6) in partially hepatectomized mice treated with adipose tissue-derived stem cell-conditioned media (CM). Mice were intravenously injected with low-glucose Dulbecco’s modified Eagle’s medium (control), lipopolysaccharide (LPS) (0.5 ng/mL), CM, or LPS-CM 1 hour after partial hepatectomy (PH) and tested for TNF-α **(A)** and IL-6 **(B)** serum levels at indicated times after PH (*P* <0.05). In each group, five mice were included per time interval (all 60 mice). Values represent mean ± standard deviation. *P <0.05.

We investigated whether the injection of CM or LPS-CM changed serum concentration of IL-6 and TNF-α in partially hepatectomized mice. Figure 3 shows that serum IL-6 and TNF-α levels peaked at day 2 after PH; however, administration of LPS-CM effectively lowered IL-6 and TNF-α levels at that time compared with the control medium as well as LPS and CM (*P* <0.05). Thereafter, these differences disappeared by day 3 after PH. IL-6 concentration at day 3 after PH, however, was still significantly lower in the LPS-CM group compared with the CM group (*P* <0.05).

### Effect of conditioned medium and lipopolysaccharide-conditioned medium on liver regeneration after PH

**Figure 4:**
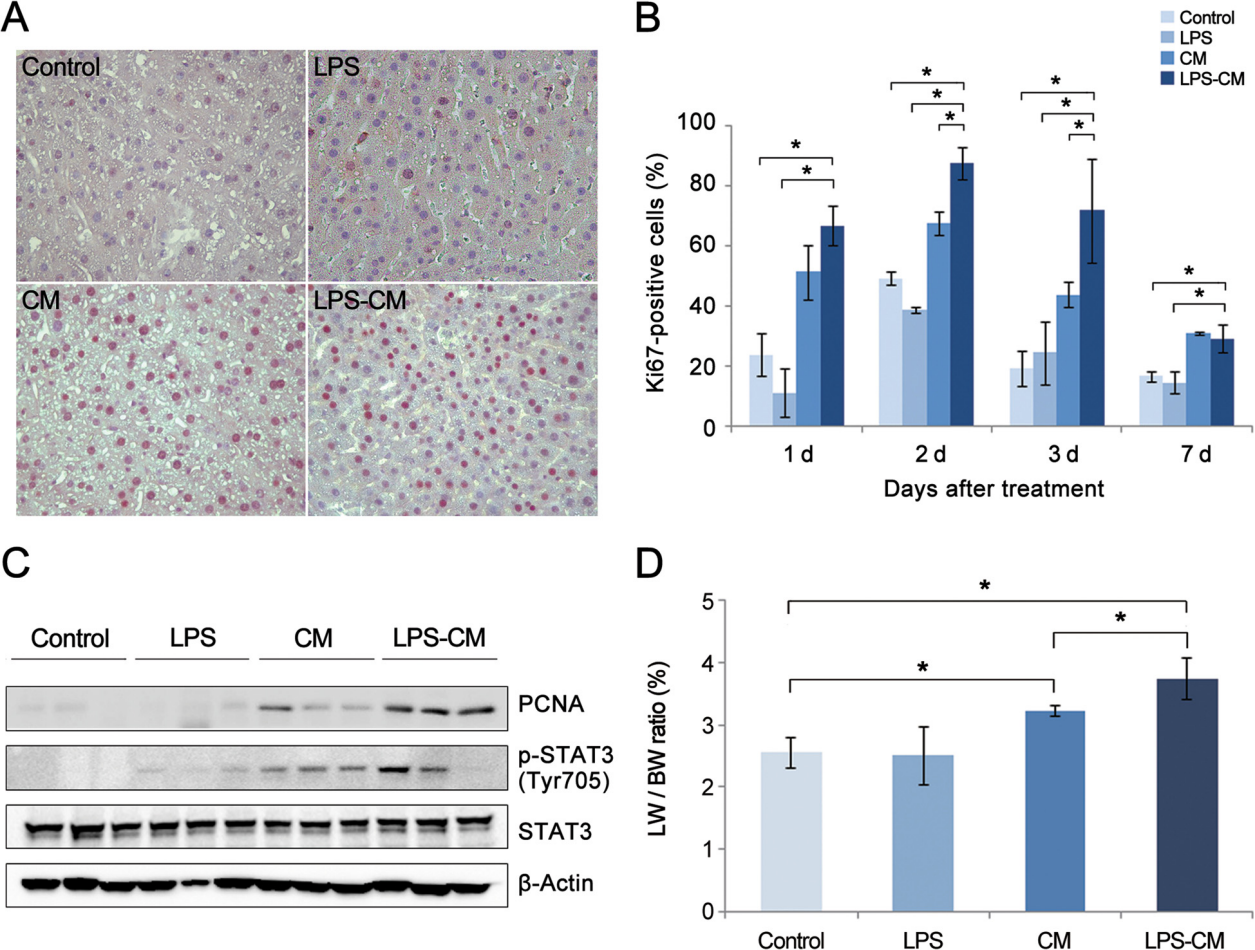
Effects of conditioned medium (CM) and lipopolysaccharide (LPS)-CM on liver regeneration in partially hepatectomized mice. Mice were intravenously injected with low-glucose Dulbecco’s modified Eagle’s medium (control), LPS (0.5 ng/mL), CM, or LPS-CM 1 hour after partial hepatectomy (PH). **(A)** Representative micrographs show liver sections stained with anti-Ki67 antibody 24 hours after PH (magnification, 400×). **(B)** The percentage of Ki67-positive cells in the liver on days 1, 2, and 3 after PH (*P* <0.05). **(C)** Western blotting of liver lysates showing that the LPS-CM group had the highest expression of PCNA and p-STAT3 on day 2 after PH. **(D)** Liver regeneration rate (percentage) based on the ratio (percentage) of liver weight to body weight (LW/BW) on day 7 after PH; the highest regeneration was observed in the LPS-CM group (*P* <0.05). *P <0.05. PCNA, proliferating cell nuclear antigen; p-STAT3, phospho-signal transducer and activator of transcription 3; STAT3, signal transducer and activator of transcription 3.

We investigated the effects of CM and LPS-CM treatment on liver regeneration in partially hepatectomized mice by (1) immunohistochemical evaluation of the number of Ki67-positive cells in the liver on days 1, 2, 3, and 7 after PH; (2) Western blotting analysis of PCNA expression on day 2 after PH; and (3) measuring liver weight on day 7 after PH (Figure 4).

**Figure 5:**
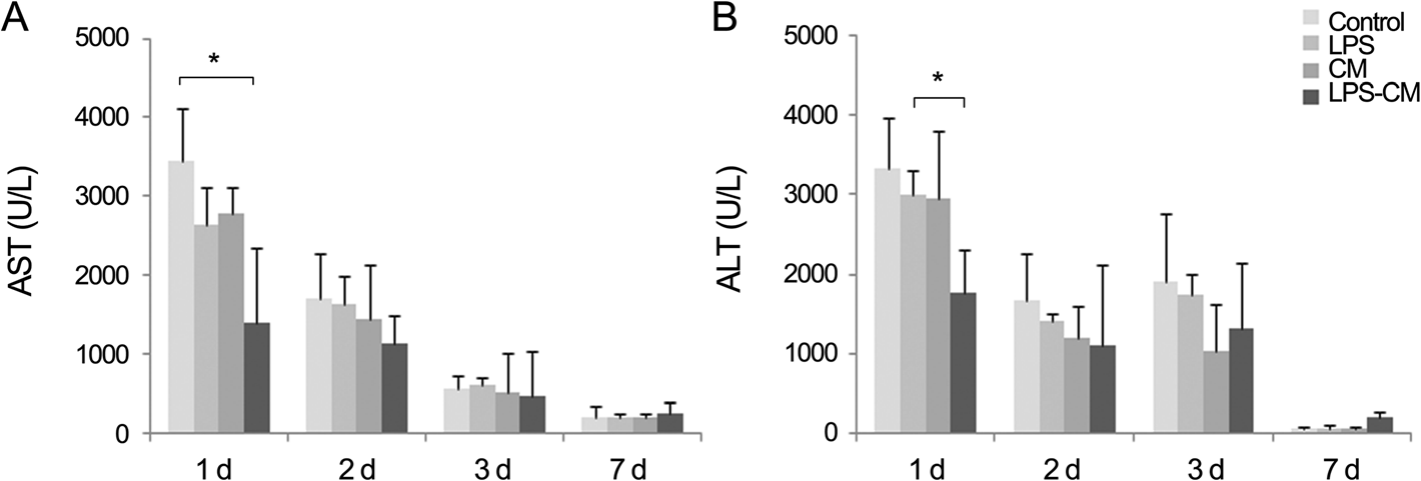
Effect of conditioned medium (CM) and lipopolysaccharide (LPS)-CM on serum levels of aspartate transaminase (AST) and alanine transaminase (ALT) in partially hepatectomized mice. Mice were intravenously injected with low-glucose Dulbecco’s modified Eagle’s medium (control), LPS (0.5 ng/mL), CM, or LPS-CM 1 hour after partial hepatectomy. At day 1 after partial hepatectomy, the LPS-CM-treated mice had significantly lower AST **(A)** and ALT **(B)** levels compared with the other groups (*P* <0.05). *P <0.05.

Antigen Ki67 is a nuclear protein associated with the transcription of ribosomal RNA and therefore is expressed exclusively in proliferating cells [40]. Figure 4A shows the representative sections of Ki67-labeled hepatocytes on day 1 after PH. The number of Ki67-positive cells peaked on day 2 and decreased thereafter in all the groups (Figure 5B). The LPS-CM mice demonstrated a significantly higher number of Ki67-positive cells in the liver compared with the control, LPS, and CM mice on day 1, 2, and 3 after PH (*P* <0.05). However, on day 7, the difference in the number of Ki67-positive cells between the CM and LPS-CM groups became insignificant.

Hepatocyte expression of PCNA is a well-established marker of liver regeneration [41]. Western blotting results indicate that the LPS-CM mice had the highest PCNA protein levels, followed by the CM group on day 2 after PH (Figure 4C). The same trend was observed for p-STAT3 expression, which was also the highest in the LPS-CM-infused mice followed by the CM-infused mice.

Finally, liver regeneration was assessed on the basis of LW/BW ratio on day 7 after PH (Figure 4D). Liver regeneration rates were calculated as 2.55 ± 0.25, 2.51 ± 0.47, 3.23 ± 0.08, and 3.73 ± 0.33 in the control, LPS, CM, and LPS-CM groups, demonstrating the highest liver regeneration in the LPS-CM group (*P* <0.05).

### Effect of conditioned medium and lipopolysaccharide-conditioned medium on liver function after PH

We evaluated serum levels of ALT and AST, the biochemical markers indicative of liver damage and hepatic dysfunction (Figure 5). We first administered LPS, CM, and LPS-CM to the control mice which did not undergo PH and identified that the solitary administration of these materials did not increase liver enzyme levels (data not shown). Next, partially hepatectomized mice were administered these materials via the tail vein. Serum concentrations of liver enzymes AST and ALT peaked at day 1 after PH and decreased thereafter, reaching near-normal levels by day 7. At day 1, the LPS-CM group showed significantly lower AST and ALT levels than the control and LPS groups (*P* <0.05), suggesting the accelerated restoration of liver function after PH.

## Discussion

In this study, it was identified that LPS preconditioning of ASCs enhanced the expression levels of the mediators, particularly involved in the inflammation (IL-6 and TNF-α) and liver regeneration (HGF and VEGF). Next, we explored the response of partially hepatectomized mice to the intravenous administration of LPS-conditioned medium (LPS-CM) in terms of inflammation, immunosuppression, hepatic function, and liver regeneration. Intravenous administration of LPS-CM reduced IL-6 and TNF-α levels more than control medium, LPS, and CM administration did. Furthermore, intravenous administration of LPS-CM enhanced liver regeneration and significantly reduced the elevated serum levels of AST and ALT. The LPS-CM group also induced higher expressions of p-STAT3 after PH than the other groups. Considering these findings, we postulated that activated IL-6/STAT3 pathway prompted by LPS-CM would be responsible for the higher liver regenerative ability in the liver. Thus, our results suggest that LPS preconditioning effectively induces ASCs to produce a secretome beneficial for hepatocyte proliferation and liver recovery.

It is widely accepted that most, if not all, cells secrete large amounts of micro- and nano-vesicles, either constitutively or upon activation signals. In our study, conditioned media, which had been obtained from ASCs, were concentrated 25-fold by using ultrafiltration units with a 3-kDa-molecular-weight cutoff. It seems that cells and larger particles were removed by sequentially increasing the centrifugal forces, and then exosomes were precipitated by centrifugation of at least 100,000 *g* for at least 2 hours. Exosomes, but also other types of microvesicles, can operate in a multitude of ways since they contain essentially biological molecules and the solutes that are present in the parental cells. Exosomes contain a number of proteins, mRNAs, microRNAs, and lipid molecules important for intercellular communication. Once secreted, exosomes can either be taken up by nearby target cells or travel to more distant sites through the blood and possibly other biological fluids. Although we did not identify exosome, we think our secretome contains a considerable amount of exosome because our way of obtaining secretome is in line with the way of obtaining exosome [12,42].

Proteomic research has identified a number of soluble proteins in the ASC secretome. Basically, mature adipose tissue itself acts as an endocrine organ releasing a wide range of regulatory proteins, such as leptin, vaspin, resistin, adiponectin, plasminogen activator inhibitor 1 (PAI-1), and pro-inflammatory cytokines [43-46], which exert hormone-like effects through the circulation [46]. ASCs have also been shown to abundantly secrete these regulatory factors, including PAI-1, placental thrombin inhibitor, pigment epithelium-derived factor, protease C1 inhibitor, and pregnancy zone protein [47-49]. A liquid chromatography-tandem mass spectrometry (LC-MS/MS) analysis of the MSC secretome has also detected 187 proteins, which included a number of mediators related with tissue repair, regeneration, and inflammation [47]. Therefore, the rationale for using stem cell secretome is exactly that it provides a unique combination of multiple regulatory factors that cannot be mechanically reproduced and should be used in totality.

In this study, we have shown that ASCs preconditioned with LPS increased the expressions of pro-inflammatory mediators, such as IL-6 and TNF-α. A large body of evidence indicates that MSCs basically possess anti-inflammatory and immunosuppressive properties [50-57]. Therefore, the reparative function of MSCs observed in so many injury models may be attributed, at least in part, to the production of paracrine factors that directly inhibit inflammatory and immune responses. However, it was also observed, though less frequently, that MSCs can induce inflammatory response [58]. In an attempt to resolve these conflicting data concerning pro- and anti-inflammatory properties of MSCs, Bunnell *et al*. [24] provided a new MSC paradigm; human MSCs, like monocytes, are polarized into two phenotypes, classified as MSC1 and MSC2 according to the downstream TLR signaling. The paradigm was based on the observation that TLR4 agonists (that is, LPS) polarized human bone marrow-derived MSCs toward a pro-inflammatory property but that TLR3 agonist (that is, poly [I:C]) polarized the MSCs toward an immunosuppressive property. Our results are in line with this proposal in that LPS, as a TLR4 agonist, polarized ASCs to show a pro-inflammatory aspect. However, ASCs were not completely polarized into the pro-inflammatory phenotype, judging from the downregulation of systemic IL-6 and TNF-α levels following LPS-CM administration. We assumed that these contrasting effects could be explained by multiple factors, including differences in the amount and duration of incubation with TLR ligands, MSC sources, and expression levels of TLR3 and TLR4.

Interestingly, LPS preconditioning of human ASCs induced higher expression of IL-6 and TNF-α in ASCs, whereas intravenous administration of LPS-CM decreased serum concentrations of mouse IL-6 and TNF-α. The surgical procedure of PH induces systemic inflammation, which involves the rise of pro-inflammatory cytokines, such as IL-6 and TNF-α. IL-6 is secreted by Kupffer cells in the liver and by monocytes and macrophages predominantly in the blood [59]. IL-6 is a pleiotropic cytokine which covers both pro-inflammatory and anti-inflammatory responses [37]. Pro-inflammatory activities of IL-6 include recruitment of inflammatory cells and inhibition of regulatory T-cell differentiation, all of which are mediated by binding to the soluble IL-6 receptor (IL-6R) [60,61]. Anti-inflammatory activities of IL-6 include STAT3-dependent regulation of hepatocyte proliferation and the induction of the hepatic acute-phase response [60,61]. These activities are dependent on the membrane-bound IL-6R. In our experiment, ASCs seems to be not completely polarized into the pro-inflammatory phenotype. Accordingly, the total composition of ASC secretome appeared to be directed toward anti-inflammation, especially in the systemic circulation. However, in the hepatic microenviroment, liver regeneration appears to be prompted by IL-6, other IL-6 family cytokines, and other mediators, all of which were derived from LPS-CM.

To the best of our knowledge, we were first to show that the LPS preconditioning of ASCs induces the release of mediators with higher liver regenerative ability; however, the exact mechanism needs to be clarified. The use of commercially available TLR4-deficient mice (C3HHEJ) is expected to contribute to the further understanding of the mechanism. It was well established that LPS triggers innate immune responses by binding to TLR4, mainly on antigen-presenting cells via nuclear factor-kappa-B (NF-κB)-dependent transcriptional events. In a report, LPS treatment to the bone marrow-derived MSCs has induced the release of mediators, such as IL-1 β, IFN-λ, and IL-6, and the activation of NF-κB via the TLR4-MyD88-dependent pathway [62]. This means that LPS-stimulated activation of NF-κB plays a critical role in the induction of pro-inflammatory cytokines from bone marrow-derived MSCs. However, further studies are required to precisely determine the signal pathway by which LPS induces ASCs to produce secretome of higher liver regenerative ability.

IL-6/STAT3 pathways play pivotal roles in regulating proliferation of hepatocytes, at least in acute liver response after hepatectomy in rodents [37,63,64]. Therefore, it can be postulated that upregulation of IL-6 and other IL-6 family cytokines has induced higher liver regeneration by the IL-6/gp130-mediated STAT3 signaling pathway. In our study, the secretome obtained from LPS-preconditioned ASCs showed the highest liver regenerative ability and expressions of p-STAT3 in both the *in vitro* and *in vivo* models. Therefore, we could think that LPS-CM promotes liver regeneration by way of IL-6/STAT3 pathway. Upon binding to membrane-bound IL-6Rs on hepatocytes, IL-6 family cytokines induce gp130 dimerization and the subsequent phosphorylation of gp130-associated Janus kinases (JAKs), which leads to STAT3 activation, followed by liver regeneration [59].

**Figure 6:**
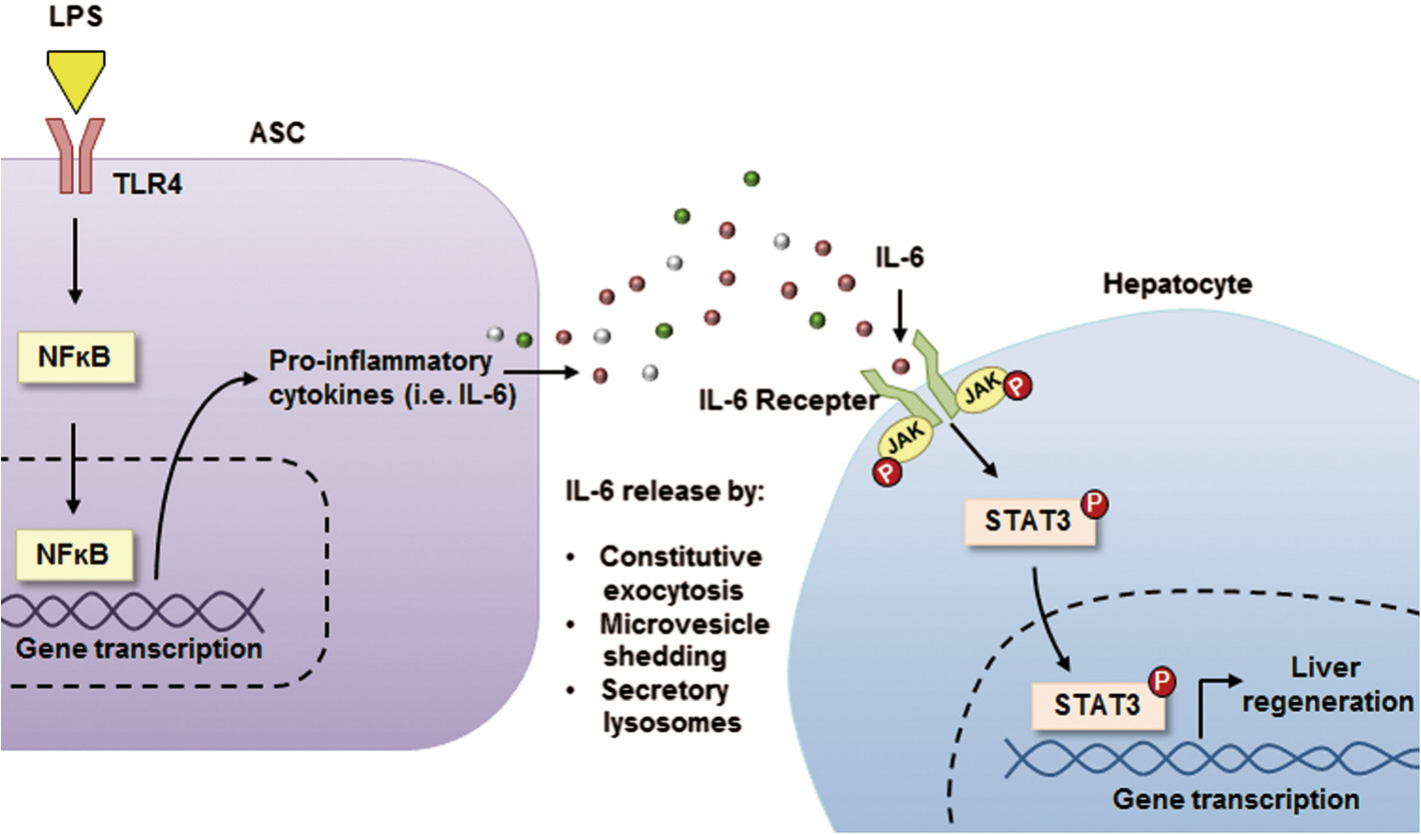
Proposed mechanisms by which lipopolysaccharide-conditioned medium (LPS-CM) induces liver regeneration in partially hepatectomized mice. It can be postulated that the LPS-preconditioned secretome releases a variety of mediators-including interleukin-6 (IL-6), tumor necrosis factor-alpha, hepatocyte growth factor, and vascular endothelial growth factor—from adipose tissue-derived stem cells (ASCs) by way of intracellular nuclear factor-kappa-B (NF-κB) activation. These mediators promote hepatocyte proliferation by way of various signaling pathways, including IL-6/STAT3 signaling. JAK, Janus kinase; STAT3, signal transducer and activator of transcription 3; TLR4, Toll-like receptor 4.

Considering these findings, we propose a possible mechanism to explain how LPS preconditioning of ASCs enhances liver regenerative ability (Figure 6). In our proposed mechanism, LPS coupled with TLR4 on ASCs induces ASCs to release pro-inflammatory cytokines, such as IL-6 and TNF-α. LPS also increases the release of the mediators with higher liver regenerative ability, such as HGF and VEGF from ASCs. The total set of molecules secreted or surface-shed by LPS-preconditioned ASCs promotes liver regeneration, especially by way of activating IL-6/STAT3 signaling pathway.

## Conclusions

We have shown that LPS preconditioning of ASCs induced the secretion of pro-inflammatory mediators with high hepatoregenerative activity, thereby maximizing the therapeutic potential of the ASC secretome. This was evidenced by the fact that LPS-CM improved the proliferative and regenerative properties of hepatocytes in both *in vitro* and *in vivo* experiments. Our results suggest that LPS preconditioning effectively stimulates ASCs to produce a secretome with the composition beneficial for liver regeneration and repair. Thus, optimizing the ASC secretome by LPS preconditioning could be a promising approach to prevent liver damage or support hepatic function or both.

## Abbreviations

ALT: alanine aminotransferase
ASC: adipose tissue-derived stem cell
AST: aspartate transaminase
CM: conditioned medium
DMEM: Dulbecco’s modified Eagle’s medium
ELISA: enzyme-linked immunosorbent assay
EV: extracellular vesicle
GAPDH: glyceraldehyde 3-phosphate dehydrogenase
HGF: hepatocyte growth factor
HRP: horseradish peroxidase
IL: interleukin
IL-6R: interleukin-6 receptor
IFN: interferon
LPS: lipopolysaccharide
LW/BW: liver weight/body weight
MSC: mesenchymal stem cell
NF-κB: nuclear factor-kappa-B
PAI-1: plasminogen activator inhibitor 1
PCNA: proliferating cell nuclear antigen
PCR: polymerase chain reaction
PH: partial hepatectomy
rpm: revolutions per minute
p-STAT3: phospho-signal transducer and activator of transcription 3
STAT3: signal transducer and activator of transcription 3
TAA: thioacetamide
TLR: Toll-like receptor
TNF-α: tumor necrosis factor-alpha
VEGF: vascular endothelial growth factor
